# Novel Therapeutic Approaches for the Treatment of Retinal Degenerative Diseases: Focus on CRISPR/Cas-Based Gene Editing

**DOI:** 10.3389/fnins.2020.00838

**Published:** 2020-08-20

**Authors:** Carmen Gallego, Manuel A. F. V. Gonçalves, Jan Wijnholds

**Affiliations:** ^1^Department of Ophthalmology, Leiden University Medical Center, Leiden, Netherlands; ^2^Department of Cell and Chemical Biology, Leiden University Medical Center, Leiden, Netherlands; ^3^Netherlands Institute for Neuroscience, Royal Netherlands Academy of Arts and Sciences (KNAW), Amsterdam, Netherlands

**Keywords:** retinal degeneration, gene editing, CRISPR/Cas systems, adeno-associated viral vectors, adenoviral vectors

## Abstract

Inherited retinal diseases encompass a highly heterogenous group of disorders caused by a wide range of genetic variants and with diverse clinical symptoms that converge in the common trait of retinal degeneration. Indeed, mutations in over 270 genes have been associated with some form of retinal degenerative phenotype. Given the immune privileged status of the eye, cell replacement and gene augmentation therapies have been envisioned. While some of these approaches, such as delivery of genes through recombinant adeno-associated viral vectors, have been successfully tested in clinical trials, not all patients will benefit from current advancements due to their underlying genotype or phenotypic traits. Gene editing arises as an alternative therapeutic strategy seeking to correct mutations at the endogenous locus and rescue normal gene expression. Hence, gene editing technologies can in principle be tailored for treating retinal degeneration. Here we provide an overview of the different gene editing strategies that are being developed to overcome the challenges imposed by the post-mitotic nature of retinal cell types. We further discuss their advantages and drawbacks as well as the hurdles for their implementation in treating retinal diseases, which include the broad range of mutations and, in some instances, the size of the affected genes. Although therapeutic gene editing is at an early stage of development, it has the potential of enriching the portfolio of personalized molecular medicines directed at treating genetic diseases.

## Introduction

Inherited retinal diseases (IRDs) constitute a heterogenous group of neurodegenerative conditions affecting the retina, a layered structure of neural origin at the back of the eye. Mutations associated with IRDs lead to retinal degeneration resulting in impaired vision and ultimately, irreversible and complete blindness (Berger et al., 2010). Clearly, this outcome negatively impacts patients’ quality of life and currently, there are no specific treatments that halt the degenerative process or, ideally, restore eyesight. Indeed, most treatments only delay the onset of symptoms or slow down the progressive retinal degeneration when applied at the early stages of the disease. Recently, the prevalence of autosomal recessive IRD variants has been analyzed and it is estimated that approximately 36% of the population carries at least one mutation that causes IRD (Hanany et al., 2020). This finding highlights the substantial global burden of blinding diseases and the imperative need for developing novel therapeutic strategies.

More than 270 genes have been identified as the cause of IRD with many disease-causing gene variants having been reported for each disorder.^1^ These variants include deletions, insertions, missense and frameshifting mutations as well as mutations that create cryptic splice sites and exonization of non-coding sequences. Genes associated with IRDs are mostly involved in photoreceptor and retinal pigment epithelium biology, including the maintenance of the visual cycle. Consequently, defective or absent retinal gene products resulting from pathological mutations invariably lead to photoreceptor death and retinal degeneration. IRDs present also different hereditary patterns (i.e., autosomal dominant, autosomal recessive or X-linked) and, in the case of recessive inheritance, patients harbor homozygous or compound heterozygous mutations (Berger et al., 2010; Khan et al., 2019). Collectively, all these parameters help defining IRDs in clinical categories attending to the genetic defect, the type of inheritance and the affected cell type, complemented with information on symptoms onset age and disease progression. IRDs can be classified in rod-dominant dystrophies (e.g., retinitis pigmentosa), cone-dominant dystrophies and generalized photoreceptor diseases (e.g., Leber congenital amaurosis). In addition, there are non-syndromic and syndromic forms of IRDs, the latter being associated with other organ pathologies. However, there is an overlap in clinical diagnoses and genotype-phenotype correlations are often difficult to establish in IRDs (Berger et al., 2010; Khan et al., 2019). A specific IRD phenotype can be caused by mutations in many different genes, one paradigmatic example being retinitis pigmentosa, associated with mutations in 84 genes. Conversely, a specific gene can be associated with several IRD phenotypes depending on the underlying mutation(s) (Berger et al., 2010; Cremers et al., 2018). Moreover, a particular genetic mutation can cause diverse phenotypes in different individuals, suggesting a role for genetic modifiers, i.e., non-allelic genetic variants, in disease penetrance and progression, which might explain the heterogeneity of certain IRDs (Venturini et al., 2012; Meyer and Anderson, 2017; Li et al., 2020).

As currently there is no standardized therapy available for patients suffering from retinal degeneration, numerous studies are focused on developing new therapeutic approaches to halt or reverse retinal degeneration. These efforts face nonetheless major challenges. Firstly, the broad range of defective genes and mutations makes it difficult to develop a single general therapy applicable to several patient groups. Rather, therapies tend to target a specific genotype. Ideally, genetic therapies for IRDs should correct or complement as many gene mutations as possible to open up the perspective for treating large patient cohorts. For example, by targeting one or more exons found to be mutational hotspots for a particular gene. Secondly, various genes causing IRDs are large genes whose coding sequences cannot be packaged in viral vectors commonly used for gene augmentation therapies (Table 1). A third challenge in the treatment of degenerative diseases, including those affecting the retina, is that the therapy needs to be applied when cells are still alive for them to be rescued from the degenerative process. Indeed, once the retina structure is compromised, and cells start dying, it is difficult to obtain any significant improvement regardless of the applied therapy. Thus, clinical research should also be focused on early diagnostics and close follow-up by the physician.

**TABLE 1:** List of large-size genes associated with inherited retinal diseases.

Gene name	Accession number	CDS (bp)	Disease(s)
*ABCA4*	NM_000350.3	6822	Recessive Stargardt disease, macular dystrophy, recessive retinitis pigmentosa, recessive cone-rod dystrophy
*CDH23*	NM_022124.6	10065	Recessive Usher syndrome, type 1d
*CEP290*	NM_025114.4	7440	Recessive Leber congenital amaurosis
*CRB1*	NM_201253.3	4221	Recessive retinitis pigmentosa; recessive Leber congenital amaurosis
*EYS*	NM_001142800.2	9435	Recessive retinitis pigmentosa
*GPR179*	NM_001004334.4	7104	Recessive complete congenital stationary night blindness
*MYO7A*	NM_000260.4	6648	Recessive Usher syndrome, type 1b
*PRPF8*	NM_006445.4	7008	Dominant retinitis pigmentosa
*RP1*	NM_006269.2	6471	Dominant retinitis pigmentosa; recessive retinitis pigmentosa
*USH2A*	NM_206933.4	15609	Recessive Usher syndrome, type 2a; recessive retinitis pigmentosa

*Representative list of genes associated with IRDs containing large coding sequences (CDS). Gene names are listed alphabetically, accession numbers and CDS sizes were retrieved from Ensembl (https://www.ensembl.org/). The disease descriptions were obtained from RetNet (https://sph.uth.edu/retnet/home.htm).*

Current experimental strategies for treating retinal degeneration can be divided in genetic therapies (i.e., gene augmentation, involving the transfer of functional wild-type copies of defective genes; and gene editing, comprising the targeted chromosomal insertion of therapeutic genes or the direct *in situ* repair of defective endogenous genes) and cell therapies (i.e., cell transplantation). In addition, next to these approaches, neurotrophic and neuroprotective factors can be delivered or modulated to prevent or slow down photoreceptor degeneration. For instance, *in vitro* studies and *in vivo* experimental therapies are exploring the use of brain-derived neurotrophic factor (BDNF), ciliary neurotrophic factor (CNTF) and glial-derived neurotrophic factor (GDNF) as candidate auxiliary therapeutic agents (Sieving et al., 2006; Jung et al., 2013; Labrador-Velandia et al., 2019). However, the benefits of this type of approaches are still limited, with some clinical studies showing no improvements over the untreated eye (Birch et al., 2016). Furthermore, some promising therapies with small molecules are moving from *in vitro* studies to clinical trials. In the case of IRDs caused by an aberrant splicing process, antisense oligonucleotides can be used to avoid the recognition of cryptic splice sites and the inclusion of pseudoexons (Collin et al., 2012; Huang et al., 2017). For other retinal disorders (e.g., retinitis pigmentosa or Leber congenital amaurosis), a pharmacological approach targeting signaling pathways is being envisioned. For instance, inhibitory analogs of the second messenger cGMP, whose signaling is relevant for the light signal transduction cascade, can inhibit the activation of cGMP effectors preventing excessive photoreceptor cell death (Vighi et al., 2018; Tolone et al., 2019). Other therapies may target the visual cycle by, for instance, assuring adequate levels of 11-*cis*-retinal, after administering the synthetic *cis*-retinoid drug QLT019001, or reducing the accumulation of toxic by-products, after applying the visual cycle modulator Emixustat for the treatment of Stargardt disease (NCT03772665) (Sundaramurthi et al., 2019). Moreover, other IRD-associated mutations result in misfolded non-functional proteins that could be rescued using small-molecule drugs or pharmacological chaperones that assure protein stability by preventing protein aggregation, and thus, cell death associated with oxidative stress (Kosmaoglou et al., 2008; Wang et al., 2016; Trachsel-Moncho et al., 2018; Lévy et al., 2019). In cases in which the disease stage is too advanced and the photoreceptor cells are irreversibly lost, optogenetic tools might be exploited to enable other retinal cells to behave as surrogate photoreceptors by endowing them with light signal transduction capabilities (Simunovic et al., 2019). In this regard, optogenetic tools based on light-sensitive proteins are being investigated in different experimental settings, including (i) *in vitro* 3D retinal organoids differentiated from induced pluripotent stem cells (iPSCs); (ii) *ex vivo* post-mortem human retinas, (iii) *in vivo* late-stage retinal degeneration murine models to determine vision restoration upon transplantation of optogenetically modified photoreceptors and (iv) *in vivo* non-human primate models to assess the safety and efficiency of adeno-associated viral (AAV) vector delivery of optogenetic tools (Chaffiol et al., 2017; Garita-Hernandez et al., 2018, 2019; Kamar et al., 2020). Finally, it is noteworthy mentioning that, beyond these experimental settings, optogenetic tools are also starting to be applied in clinical trials (e.g., NCT03326336, for retinitis pigmentosa patients).

These candidate therapeutic solutions for treating the eye have been recently reviewed from different perspectives (see, e.g., Scholl et al., 2016; Dias et al., 2018; Sanjurjo-Soriano and Kalatzis, 2018; Vázquez-Domínguez et al., 2019). Here, we will focus on covering the equally fast-paced advances in the gene editing field, with an eye for its potential to the management and treatment of congenital ophthalmologic disorders. We start by briefly discussing “classic” gene augmentation approaches and their limitations and, thereafter, move forward by elaborating on key developments that gene editing techniques are currently undergoing. In the context of these genetic interventions, we also discuss the different vectors and delivery options to reach retinal cell types, including the potential of high-capacity adenoviral vectors (HC-AdVs) to deliver large therapeutic transgenes or gene editing tools to retinal cells.

## Genetic Therapies for Treating IRDs: From Gene Augmentation to Gene Editing

Genetic therapies consist of providing new genetic information into cells damaged by trauma, infectious agents or inherited diseases with the goal of attaining a stable therapeutic effect. Monogenic inherited disorders constitute an ideal target for genetic therapies involving either “classical” gene therapy based on gene addition or, more recently, therapeutic gene editing based on site-specific genome modifications. This stems from the fact that the disease-causing gene is known with its mechanistic function being often well understood. Gene therapy encompasses the delivery of “healthy” wild-type versions of mutant alleles; whereas gene editing entails the transfer of molecular tools (e.g., sequence-specific programmable nucleases) needed to inactivate or correct disease-causing mutant alleles (DiCarlo et al., 2018; Takahashi et al., 2018; Gordon et al., 2019).

Depending on the inheritance pattern of the disease, the therapeutic approach varies. Gene therapies involving gene addition or augmentation can be used for the complementation of homozygous recessive loss-of-function mutations through the delivery and expression of a “healthy” functioning copy of the affected gene. The delivery of the genetic material to the retina can be done in different ways and these will be discussed in the next section. Currently, AAV vectors are the predominant candidate therapeutic options for tackling ocular diseases with a gene therapy to treat Leber congenital amaurosis based on the delivery of *RPE65* by an AAV serotype 2 (AAV-2) vector having already been approved by the Food and Drug Administration (United States) and the European Medicines Agency (Russell et al., 2017). Long-term efficacy of AAV-based *RPE65* gene therapies appeared to be limited to 2 years after treatment (Wang et al., 2020), however, a recent follow-up report on therapeutic outcomes confirmed that 86% of patients receiving this approved *RPE65* gene therapy (named voretigene neparvovec) maintained improved visual function 4 years after the treatment (Drack et al., 2019). More results are warranted as the study includes a 15-year follow-up of patients (NCT00999609).

The small cargo capacity of AAV capsids (∼4.7 Kb) makes these viral vectors incompatible with the delivery of large transgenes. Various IRD-associated genes have coding sequences that exceed the maximum packaging capacity of AAV capsids (e.g., *ABCA4*, *CEP290*, and *USH2A*) (Arbabi et al., 2019; Table 1). Thus, other approaches are needed for treating IRDs caused by such genes. Gene editing, while offering the potential for correcting *in situ* endogenous mutated alleles, can also be applied to diseases caused by gain-of-function allelic mutations that exert dominance over wild-type alleles (e.g., mutations in *RHO* gene responsible for autosomal dominant retinitis pigmentosa). In these cases of dominant inheritance, gene editing tools can be designed for (i) the specific inactivation of mutant, disease-causing, alleles, (ii) restoring the endogenous allele or (iii) inserting an exogenous “healthy” copy at a specific genomic position, e.g., at “safe harbor” locus “from where transgene overexpression can ensue”.

### Genome Editing: Advances in CRISPR/Cas-Based Gene Editing Systems for Non-dividing Cells

In this section, we focus on recent advancements in the conversion of clustered regularly interspaced short palindromic repeats (CRISPR)/CRISPR-associated Cas (CRISPR/Cas) nuclease systems into gene editing tools, in particular, for the precise insertion of genetic material in post-mitotic cells. CRISPR/Cas systems provide adaptive immunity in prokaryotes (Barrangou and Marraffini, 2014). In 2012, it was found that CRISPR-derived nucleases cleave DNA in an RNA-programmable manner involving initial protein-DNA and subsequent RNA-DNA interactions (Gasiunas et al., 2012; Jinek et al., 2012). On the basis of these seminal findings, the native CRISPR/Cas9 system from *Streptococcus pyogenes* was the first to be readily adapted into RNA-guided nucleases (RGNs) for genome engineering in mammalian cells and tissues (Cho et al., 2013; Cong et al., 2013; Jinek et al., 2013; Mali et al., 2013). It consists of an RNA-guided Cas9 enzyme with two separate endonuclease domains (i.e., HNH and RuvC). After Cas9 binding to a so-called protospacer adjacent motif (PAM), hybridization of the 5’ end (spacer) of the single guide RNA (gRNA) to a typically 20-nt complementary sequence (protospacer) activates the nuclease domains leading to the cleavage of the target DNA sequence at both strands (Doudna and Charpentier, 2014). The gRNA consists of a fusion between two key components derived from the native CRISPR locus, i.e., the sequence-specific CRISPR RNA (crRNA) and the Cas9 scaffolding trans-activating crRNA (tracrRNA) (Doudna and Charpentier, 2014). There are different types of CRISPR endonucleases that vary in their PAM sequence and spacer length requirements as well as in their specificity and type of DNA cleavage products, i.e., blunt-ended or staggered double-stranded DNA breaks (DSBs). Currently, the most commonly used RGNs contain the Cas9 proteins from *Streptococcus pyogenes* (Doudna and Charpentier, 2014) or *Staphylococcus aureus* (Ran et al., 2015), whose PAMs read NGG or NNGRRT, respectively.

RGN complexes have been used in a great variety of organisms (e.g., zebrafish, insects and mammals). They offer flexibility and are much easier to construct and customize than previous gene editing tools such as transcription activator-like effector nucleases (TALENs) and zinc-finger nucleases (ZFNs) (see, e.g., Chandrasegaran and Carroll, 2016; Yanik et al., 2017). As with TALENs and ZFNs, RGNs can also present off-target activities that constitute a major drawback for their application in the clinic. Thus, variants of Cas nucleases have been engineered to limit these harmful off-target effects and hence improve the specificity while, for the most part, maintaining the efficiency of the corresponding RGNs (Nakade et al., 2017; Chen and Gonçalves, 2018). For example, high-specificity nuclease variants based on amino acid substitutions (Kleinstiver et al., 2016a; Slaymaker et al., 2016) or sequence- and strand-specific nucleases (nickases) that only produce a single-stranded cut in the DNA (Jinek et al., 2012; Cong et al., 2013; Mali et al., 2013).

**FIGURE 1:**
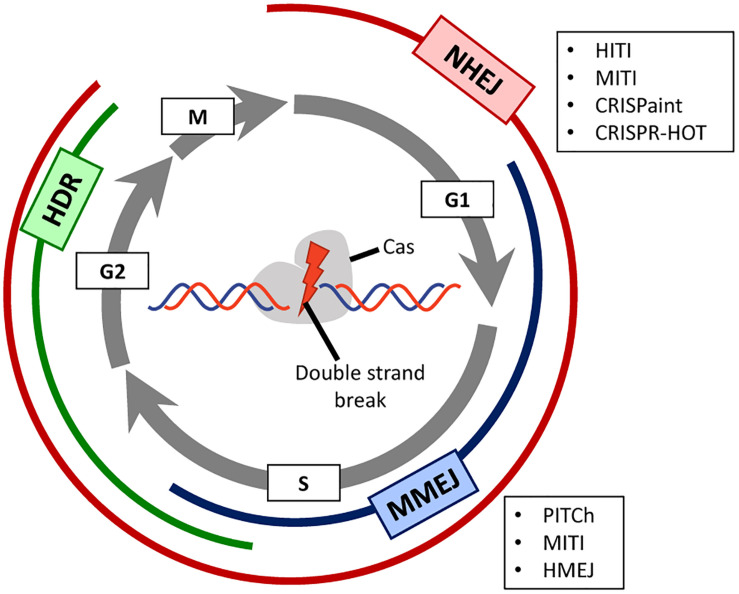
Prevalence of DNA repair pathways during the different stages of the cell cycle. A Cas-containing RGN is directed to a target site in the genome consisting of a PAM and a sequence complementary to the spacer portion of the guide RNA. Depending on the RGN type, the produced double-stranded DNA breaks (DSBs) present either blunt or staggered ends. Depending on the phase of the cell cycle (displayed as gray circular arrows), different DNA repair mechanisms, each of which with their own set of factors, will be activated for repairing the site-specific DSB. Non-homologous end joining (NHEJ, in red) is active during the whole cell cycle except in mitosis, and homology-directed repair (HDR, in green) is mainly active during the S/G2 phases. Microhomology-mediated end joining (MMEJ, in blue) is an alternative NHEJ pathway mainly active during the G1/early S phases. This pathway shares some features with canonical NHEJ and HDR but uses a different molecular machinery and short homology sequences to repair the DNA break. In post-mitotic cells, the mostly error-free, HDR pathway is not active. For this reason, there are several innovative CRISPR/Cas-based strategies (boxed acronyms) that rely instead on NHEJ or MMEJ to allow for precise gene knock-in in non-dividing cells.

The catalytic activity of the Cas9 endonuclease produces a DSB in the target DNA region complementary to the gRNA spacer sequence. DSBs are detected by the DNA damage response and repaired by different DNA repair pathways whose selection is, to a great extent, dependent on the cell cycle phase (Ceccaldi et al., 2016; Yeh et al., 2019; Figure 1). The non-homologous end-joining (NHEJ) pathways (canonical and alternative) are active during most phases of the cell cycle and are generally considered more error-prone mechanisms than those underlying homology-directed DNA repair (HDR) pathway. In the process of end-to-end joining of chromosomal termini, NHEJ pathways can introduce small insertions and deletions (indels) especially in the presence of a programmable nuclease that re-cleaves accurately joined termini until an indel disrupts its target sequence. Usually, the activation of NHEJ pathways by programmable nucleases is a key strategy to generate target gene knock-outs upon the incorporation of frameshifting indels within coding sequences (Chandrasegaran and Carroll, 2016). The HDR pathway involving homologous recombination comprises extensive DSB end-processing, single-strand DNA invasion, donor-templated DNA synthesis and Holliday junction resolution. This HDR pathway is only active during the S and late G2 phases of the cell cycle being hence restricted to dividing cells (Iyama and Wilson, 2013; Figure 1), and relies on exogenous donor DNA templates containing regions homologous to sequences surrounding the DSB. The HDR mechanism is harnessed to direct precise genome editing and generate specific targeted knock-ins whose lengths can vary from single base pairs to whole transgenes (Doudna and Charpentier, 2014; Chandrasegaran and Carroll, 2016; Chen and Gonçalves, 2018; Yeh et al., 2019). A third mechanism for DNA repair is microhomology-mediated end-joining (MMEJ) (McVey and Lee, 2008), which is a non-canonical NHEJ pathway that relies on microhomology regions (5–25 base pairs) surrounding the DSB. This DNA repair pathway is usually active in G1/early S phases (Figure 1).

There are several studies in the field of retinal degeneration that have explored the feasibility of applying CRISPR/Cas9-based gene editing tools *in vivo* to inactivate dominant alleles through the NHEJ pathway. A first proof-of-concept work, in which expression of the yellow fluorescent protein reporter was reduced in the retina of transgenic mice (Hung et al., 2016), paved the way for other researchers starting to investigate the potential of engineered CRISPR/Cas systems for disrupting or correcting gene variants associated with IRDs. For instance, Bakondi et al. (2016) achieved the disruption of a *Rho* allele harboring a dominant mutation in a rat model of retinitis pigmentosa. The strategy induced indels in the mutant allele but not in the wild-type allele and, in doing so, prevented retinal degeneration that subsequently led to improved visual function (Bakondi et al., 2016). In another study, a deletion of an intronic fragment of the murine *Cep290* gene, homologous to the human region that contains a deep intronic variant causing Leber congenital amaurosis type 10, was achieved in mouse retinas (Ruan et al., 2017). These studies used AAV vectors to deliver RGNs directly to mouse retinas and will be further discussed below.

**FIGURE 2:**
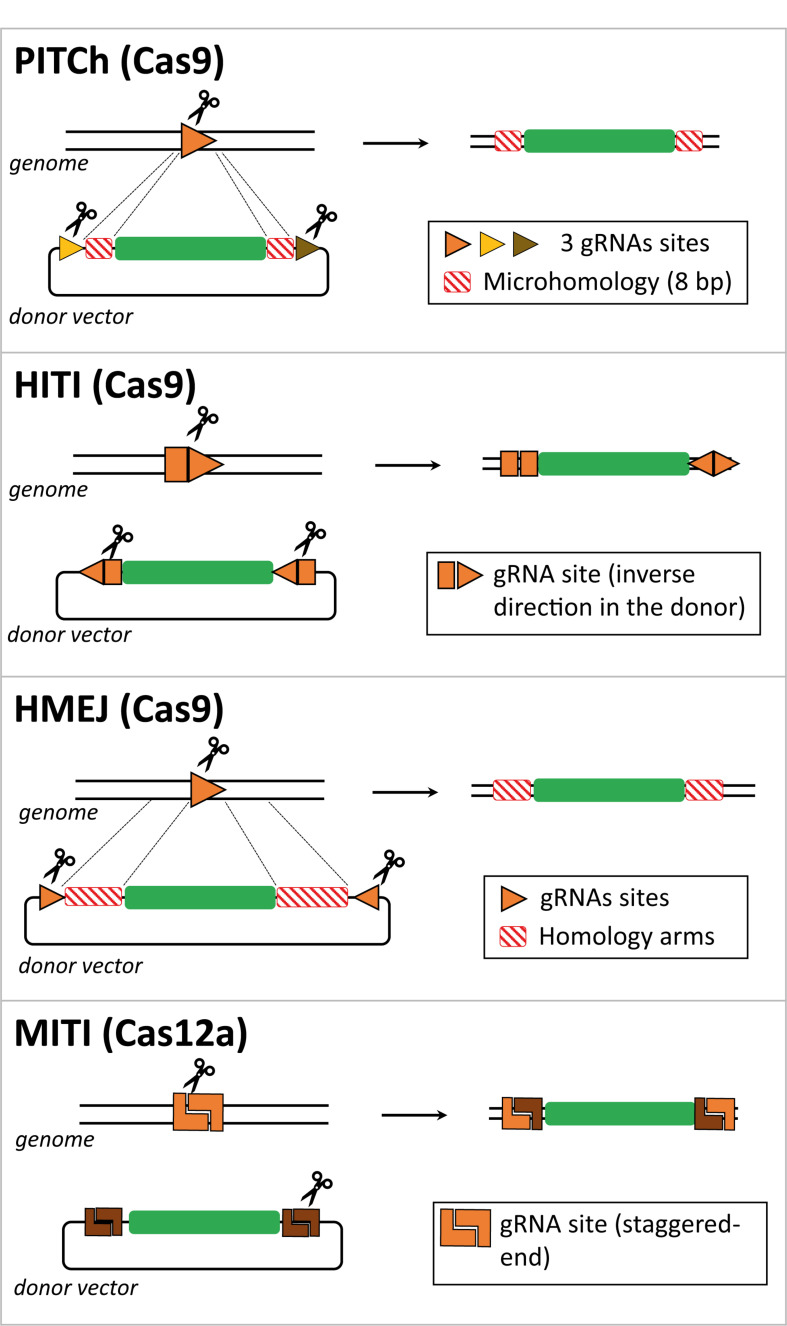
Comparison between CRISPR/Cas-based strategies for targeted gene insertion in non-dividing cells. Schematic representation of several gene editing methodologies developed to direct targeted transgene integration in the genome independently of the HDR pathway, that is not active in non-dividing cells such as photoreceptors. The genomic locus and the donor DNA are cut by Cas effector proteins at gRNA target sites. Typically, the donor DNA contains two gRNA target sites to release the exogenous genetic information from the context of the delivery vector and aid its recombination with the target locus. These two gRNA target sites can have identical or dissimilar sequences and can be incorporated in a direct or inverted orientation. The Cas-induced DSB can generate microhomology regions between target and donor DNA, e.g., in the case of the PITCh and MITI methods. In the case of the HMEJ method, the lengths of the homology regions in the donor DNA are similar to those incorporated in HDR donors (i.e., ∼800 bp), yet they can be shortened to 24–48 bp.

Importantly, the transcriptional signatures of the DNA repair pathways in the retina have been recently characterized using transcriptomic analyses of *in vivo* and *in vitro* models (Pasquini et al., 2020). This study showed that photoreceptors, as post-mitotic cells, display low expression levels of HDR pathway components; whereas NHEJ and MMEJ pathway factors are expressed and active in these cells. Given that precise gene editing has generally relied on HDR, there are continuous efforts aimed at improving the efficiency of seamless DSB-mediated gene editing through the activation and recruitment of alternative DNA repair pathways that remain active in terminally differentiated cells, such as those that constitute the retina in mammals. Crucially, in the past few years, there has been a myriad of novel CRISPR/Cas-based strategies that allow for the precise insertion of genetic material at a specific genomic locus in non-dividing cells and, following from the above, these efforts are particularly relevant for the treatment of retinal disorders (Nami et al., 2018; Broeders et al., 2020). These new strategies are also dependent on the delivery of a donor template that is designed for the site-specific insertion of exogenous genetic material into the genome; yet the insertion process takes place independently of the homologous recombination mechanism. In the next paragraphs, we will describe these new approaches and how they can be exploited to target the retina (Figure 2).

### Novel Strategies for Precise Gene Targeting in Non-dividing Cells

A homologous recombination-independent gene editing strategy is named Precise Integration into Target Chromosome (PITCh) and it was initially described as an alternative for the more laborious process of designing and incorporating extensive homology regions required in HDR donor templates. This method was designed to be used with both TALENs and RGNs and it depends on the engagement of the MMEJ pathway in that the programmable nuclease-induced DSBs at donor and target sequences create microhomology regions that allow for site-specific chromosomal integration of the transgene independently of long stretches of homology (Figure 2; Nakade et al., 2014; Sakuma et al., 2016). However, in these proof-of-principle experiments, in which an exogenous reporter cassette was inserted in the last exon of the human fibrillarin (*FBL*) gene, were only performed in cultures of transformed human embryonic kidney (HEK) 293 cells and no quantitative assessments were made (Nakade et al., 2014). Furthermore, as expected from the MMEJ pathway, indels were found at the junctions between the endogenous DNA and the integrated expression cassette, although the reading frame was not affected and the fluorescent reporter protein was expressed (Nakade et al., 2014).

Suzuki et al. (2016) designed an Homology-Independent Targeted Integration (HITI) strategy to direct the integration of a transgene through the RGN-induced activation of the NHEJ pathway. In this case, the designed donor DNA includes RGN target sites flanking the transgene, which are in the reverse orientation to that found in the genomic locus (Figure 2). Upon RGN-mediated cleavage of both genome and donor sequences, DNA ends are ligated by NHEJ. In instances in which the transgene is integrated in an inverted orientation, the gRNA target sites will be reconstructed, becoming available for further cleavage and excision from the genomic locus, giving another opportunity for correct integration. Once the transgene is inserted in the appropriate orientation, the gRNA target sites are disrupted, preventing further RGN cleavage and unwarranted donor DNA excision (Suzuki et al., 2016, 2018). The HITI process was applied *in vitro* in non-dividing cells, specifically in cultures of primary murine neurons and human embryonic stem cell-derived neurons, albeit the knock-in efficiency was low (i.e., 0.58% of absolute gene targeting efficiency in primary mouse neurons). Of note, the PITCh approach barely yielded knock-in events in cultured neurons (Suzuki et al., 2016). To increase the *in vivo* efficiency of HITI, the authors used AAV vectors to deliver RGNs and donor constructs rather than performing electroporation. By injecting AAVs in the visual cortex of the adult mouse brain, they reached an absolute knock-in efficiency of 3.5% compared to the 0.2% achieved by *in utero* electroporation. Suzuki and colleagues also tested the AAV-HITI method in the Royal College of Surgeons rat model of retinitis pigmentosa, in which a deletion encompassing part of intron 1 and exon 2 sequences of the *Mertk* gene leads to dysfunctional retinal pigment epithelium and subsequent retinal degeneration. This data is highly relevant for the topic of this review, as the successful integration of the full *Mertk* exon 2 sequence in the genome via the subretinal delivery of two AAV vectors, one containing the HITI donor construct and the other the RGN expression unit, significantly improved retinal degeneration and eye function (Suzuki et al., 2016). These promising results warrant additional preclinical studies aiming at treating retinitis pigmentosa and other retinal degeneration disorders, especially in models other than rodents which are more representative of human phenotypes. Furthermore, the use of AAV vectors is limited whenever gene editing interventions depend on the knocking-in of large genetic payloads and/or large RGN components, such as, *S. pyogenes* Cas9 and multiplexing gRNA pairs designed for targeted chromosomal deletions (Cong et al., 2013).

Another genome engineering method, dubbed Homology-Mediated End-Joining (HMEJ), was developed by Yao and colleagues, in which the donor DNA, similarly to HDR constructs, contains homology regions flanking the transgene. In the case of HMEJ donors, however, the homology regions are further flanked by gRNA target sites to allow for the excision of the transgenic sequences (Yao et al., 2017; Figure 2). Repair of the DSBs, induced at the genomic site and the donor DNA, can in principle be achieved via HDR or NHEJ repair pathways, although the authors did not investigate which DNA repair mechanisms underlie the HMEJ methodology. The performance of HMEJ-based gene editing in terms of targeted gene knock-in efficiencies varied depending on the experimental system. When comparing gene knock-in efficiencies, there were no differences between HMEJ- and HDR-based methods in mouse embryonic stem cells and in a murine neuroblastoma cell line (approximately 7% and 30% of absolute knock-in frequencies, respectively) (Yao et al., 2017). However, in the human HEK293T cell line and in murine primary neurons, HMEJ resulted in higher relative gene knock-in frequencies when compared to those resulting from the HDR-dependent strategy (approximately 2.6 to 4 fold increase) (Yao et al., 2017). The HMEJ approach was further tested to modify mouse embryos yielding, in this system, gene knock-in levels that were higher than those achieved by gene editing approaches based on the engagement of the NHEJ, HDR or MMEJ pathways. In addition, similarly to the study by Suzuki and colleagues (Suzuki et al., 2016), AAV vectors were used to deliver *S. pyogenes* RGNs and, in this case, HMEJ donor constructs to the visual cortex of the adult mouse brain. The authors reported an absolute gene knock-in efficiency of 5.3% (Yao et al., 2017). This innovative strategy holds therapeutic potential as it was also used for *in vivo* correction of an hereditary tyrosinemia disorder caused by mutations in the *Fah* gene, coding for fumarylacetoacetate hydrolase, that results in liver failure (Grompe et al., 1993; Yao et al., 2018). The HMEJ-based gene editing method successfully rescued *Fah* expression with hepatocyte proliferation being higher than that obtained with the MMEJ-based method (Yao et al., 2018). In another study, the HMEJ strategy was compared side-by-side with the HITI- and HDR-based gene editing methods in chicken DF-1 and primordial germ cells. The results from this work showed that HMEJ was the most efficient in mediating precise gene knock-in (Xie et al., 2019). A recent publication introduced another HMEJ-based approach (named GeneWeld) in which shorter homology segments of 24–48 base pairs were sufficient to drive precise reporter gene knock-in in zebrafish and mammalian cells using easily engineered donor DNA constructs in which the reporter transgene was flanked by universal gRNA target sequences (Wierson et al., 2020). Although more mechanistic insights are needed to understand how HMEJ operates, these studies expand nonetheless the range of donor DNA toolboxes and associated CRISPR/Cas-based gene editing strategies. To enrich the options to generate reporter cell lines and animal models, the CRISPaint system was developed to introduce reporter tags at the C-terminal coding portion of any gene of interest using a universal donor plasmid and the NHEJ pathway to repair the RGN-induced DSBs (Schmid-Burgk et al., 2016). More recently, this methodology was applied in human organoids, named CRISPR-HOT for homology-independent organoid transgenesis (Artegiani et al., 2020).

As previously mentioned, Cas9 proteins from the type II CRISPR systems of *S. pyogenes* and *S. aureus* are the most commonly used Cas effector proteins. Importantly, Cas orthologs have been identified in many other species of bacteria and have been further adapted for genome engineering of mammalian cells (Nakade et al., 2017; Chen and Gonçalves, 2018; Ishino et al., 2018). For instance, Cas12a from the type V CRISPR system of *Acidominococcus* sp., formerly known as Cpf1, is an RNA-guided endonuclease that differs from Cas9 in that it naturally has a single guide RNA with a strand polarity opposite to that of the composite single gRNA of Cas9 proteins formed by a fusion between crRNA and tracrRNA moieties. Other differences between RGNs based on Cas12a and Cas9 nucleases include the generation by the former of staggered ends instead of blunt ends and the recognition of T-rich instead of G-rich PAMs (Zetsche et al., 2015). It is also noteworthy mentioning that Cas12a RGNs present higher specificities than their Cas9 counterparts (Kleinstiver et al., 2016b). Moreover, similarly to Cas9, novel Cas12a variants have been engineered through mutagenesis screens to alter PAM preferences and hence enlarge the range of targetable sequences by the corresponding RGNs (Gao et al., 2017). Altogether, these features make Cas12a an appealing alternative for precise gene editing. Indeed, a recent study has reported an innovative Cas12a-based method named microhomology-dependent targeted integration (MITI) that permitted increased gene knock-in efficiency when compared to the Cas9-based HITI method (Li et al., 2019). This methodology is based on the generation of complementary sticky sequences between transgene and target site termini that assist in directing genomic DNA integration (Figure 2). This proof-of-concept study was only performed in cultured cell lines and requires further optimization to assure seamless transgene integration at the intended genomic loci. Indeed, the authors reported that approximately 30% of the integration events were not accurate at the 5’ junction with the precision observed at the 3’ junction being even less accurate (Li et al., 2019). These outcomes were partially improved by incorporating two different Cas12a gRNA cleavage sites flanking the transgene resulting in higher integration accuracy at the 3’ junction, although at the 5’ junction, 36.6% of the integration events were still inaccurate. Notwithstanding the fact that MITI facilitates site-specific knock-ins, the use of several gRNAs complicates somewhat its application, including in the context of candidate genetic therapies. In this regard, a platform for Cas12a-based multiplexed genome editing, named SiT-Cas12a, has been developed based on a single transcript that encodes for Cas12a from *Acidominococcus* sp. and an array of crRNAs under the control of a single promoter (Campa et al., 2019). This strategy was used to target multiple sites in a gene, resulting in knock-out frequencies higher than those achieved by targeting a single site (37% versus 11% of gene knockouts in cell populations, respectively). This approach might further empower the capabilities of CRISPR/Cas techniques to investigate the underlying mechanisms of multifactorial diseases.

In conclusion, a plethora of innovative tools and strategies are being devised to improve the performance of CRISPR/Cas-based genomic manipulations and, as a result, increase the range and potential of therapeutic gene editing applications. These various reagents and methods are highly relevant for designing new candidate genetic therapies and, as a consequence, increasing the chances of advancing pre-clinical proof-of-concept studies into much-needed effective ophthalmologic treatments. The capacity for achieving targeted integration of transgenes in post-mitotic cells (e.g., photoreceptors), offered by several of the covered gene editing approaches, might also aid in providing mechanistic insights into retinal diseases and hence guide the development of genetic therapies for these conditions.

## Concerns and Challenges for the Use of Gene Editing in the Treatment of Retinal Diseases

Before making the transition toward clinical applications, gene editing techniques must reach higher efficiency and safety profiles. These parameters are contingent upon the delivery and specificity of the necessary gene editing reagents as well as on their tight control once inside the cell (Yu and Wu, 2018). As described in the previous section, several innovative strategies have been developed that in principle might allow for precise gene insertion in post-mitotic cells such as those affected by congenital disorders of the retina. However, it is important to highlight that most techniques were developed and tested in *in vitro* systems with only a few of them having, so far, made the transition to *in vivo* genome editing testing in physiologically relevant animal models.

A major challenge concerns the need for efficient delivery of gene editing tools into the proper target cells *in vivo* and subsequently achieve gene modification in a sufficient number of cells for preventing or halting disease progression. The delivery to target cells can, to some extent, be controlled by the choice between subretinal or intravitreal injection. Undertaking multiple injections might increase the percentage of cells that uptake the genetic therapy. However, the evaluation of these parameters is limited to end-point functional readouts as taking retina biopsies from treated patients to quantify the percentage of modified cells is not feasible. Neither is tracking or selecting cells for *in vivo* assessment of the genetic therapy. In addition, a balance between increased efficacy and adverse side effects must be carefully established. One may argue that the risks associated with multiple injections (e.g., retina detachment or bleeding) are non-negligible. As for other neurodegenerative disorders, it is also crucial to determine the optimal time of intervention or therapeutic window. Early diagnostics and treatment undoubtedly generate better clinical outcomes. Further pre-clinical studies using animal models could shed some light into the genuine efficacy of genetic therapies at later stages of degeneration that resemble the advanced degenerative phenotype of some patient populations.

Moreover, continuous expression of RGNs raises safety issues because it can cause cytotoxicity or trigger immune responses due to their foreign, prokaryotic, origins. *In vitro* transcribed gRNAs can trigger innate immune responses and anti-Cas9 antibodies (against *S. pyogenes* and *S. aureus*) together with Cas9-specific T cells have been detected in the blood of healthy human donors (Crudele and Chamberlain, 2018; Kim et al., 2018; Charlesworth et al., 2019; Mehta and Merkel, 2020). Preexisting adaptive immunity, through cytotoxic T cells, might be responsible for the killing of cells expressing Cas9 antigens. The immune privileged status of the retina at early stages of degeneration and/or the administration of immunosuppression treatments might ameliorate or overcome these adverse reactions. Importantly, the safety, tolerability and efficacy of a CRISPR/Cas9-based therapy will be addressed in a recent, currently recruiting, Phase I/II clinical trial to treat patients with Leber congenital amaurosis type 10 (NCT03872479). In this study, patients will receive the EDIT-101 therapy, an AAV5 vector expressing Cas9 from *S. aureus* and two gRNAs that target and remove a deep intronic variant in the *CEP290* gene, aiming at restoring normal gene expression and ensuing functional rescue (Maeder et al., 2019). In addition, an ideal CRISPR/Cas-based genetic therapy should include a strategy to shut down the machinery once the editing process is over, to decrease the chances for off-target activities. For example, optogenetic-regulated switches could be an interesting approach to regulate transgene expression (Polesskaya et al., 2018). Indeed, viable strategies for spatiotemporal control of the CRISPR/Cas components should ideally be envisioned and tested before applying gene editing as a therapy for retinal degeneration.

### Genome Editing Approaches Independent of Double-Stranded DNA Break Formation

Another safety issue arises directly from RGN-induced DSB formation in that DSBs are one of the most severe types of DNA lesions as they can lead to deleterious rearrangements in the genome, e.g., intra- and inter-chromosomal translocations. For this reason, nickases are becoming increasingly investigated for bringing about precise HDR-mediated seamless gene editing as single-stranded DNA breaks are intrinsically less disruptive to the genome when compared to DSBs (Chen et al., 2017, 2020; Nakajima et al., 2018; Hyodo et al., 2020). Furthermore, in order to use CRISPR/Cas-based techniques for genetic therapies, a deep knowledge on how DNA repair pathways operate in retinal cell types is needed. For instance, favoring certain NHEJ pathways (e.g., MMEJ) might lead to an increase in mutagenic events. Pre-clinical studies should aim at carefully understanding which mechanisms are prevalently involved in the repair of RGN-induced DSBs resulting in transgene integration, while minimizing the generation of mutagenic indels at target and off-target sites. The further development of sensitive and unbiased genome-wide DSB detection methods should assist and complement the development of bioinformatic tools that can predict with higher confidence off-target events. Together these technologies should be instrumental for monitoring and validating the specificity of gene editing procedures.

Importantly, alternative CRISPR/Cas-based genome editing strategies that do not require DSB formation or donor DNA templates are being engineered and investigated (Komor et al., 2016; Nishida et al., 2016; Chen et al., 2017, 2020; Gaudelli et al., 2017; Nakajima et al., 2018; Anzalone et al., 2019; Hyodo et al., 2020). One of such alternative strategies is base editing. The basic principle of base editors consists of fusing a Cas9 nickase to a cytosine or adenine deaminase that induces single base pair changes (i.e., C > T or A > G transitions, respectively) within a so-called editing window located in the protospacer bound by the gRNA spacer (Komor et al., 2016; Gaudelli et al., 2017; Rees et al., 2017). The first base editors contained the cytosine deaminases APOBEC1 from rat (Komor et al., 2016) or PmCDA1 from sea lamprey (Nishida et al., 2016). Subsequently, base editors capable of inducing A > G transitions were obtained through directed evolution and selection of a bacterial transfer RNA adenosine deaminase that acts on DNA (Gaudelli et al., 2017). More recently, a dual base editor has been engineered by fusing a Cas9 nickase to both an adenine and cytosine deaminase base editors, allowing simultaneous substitutions in adenine and cytosine bases in the same target sequence (Grünewald et al., 2020). Although base editors can function in non-dividing cells, they have a limited performance in terms of the range of nucleotide conversions that they can implement. Indeed, base editors install neither transversions nor small insertions or deletions. Importantly, recently, prime editing has expanded the repertoire of DSB-free CRISPR/Cas-based systems for gene editing in that it permits installing all possible base-to-base conversions as well as small insertions and deletions (Anzalone et al., 2019). This new methodology is based on a nickase Cas9 fused to an engineered reverse transcriptase (RT). Cas9 is directed to the target sequence with a prime editing guide RNA (pegRNA) consisting of a 3’ end-extended gRNA that acts as a primer for reverse transcription initiation. Upon site-specific nicking of the PAM-containing strand, the released single-stranded DNA hybridizes to a portion of the pegRNA (primer binding site) that provides for a free 3’-OH group for reverse transcription of the desired genetic modifications encoded in another portion of pegRNA (RT template). Ultimately, the synthesized complementary DNA (cDNA) becomes incorporated at the target site after a series of events presumably encompassing genomic DNA-cDNA hybridization and non-hybridizing DNA flap removal. The prime editor is a versatile tool in that it is independent of donor DNA templates and, as aforementioned, allows for the introduction of any base-pair substitution, small deletions (i.e., up to 80-bp) and small insertions, e.g., epitope tags (Anzalone et al., 2019). Interestingly, although the highest efficiencies of prime editing were observed in transformed HEK293T cells, the authors demonstrated that post-mitotic cells, in particular primary cortical murine neurons, can also undergo prime editing (Anzalone et al., 2019). Clearly, further research is warranted to improve the efficiency of prime editing, assess its genome-wide off-targets and its performance in *in vivo* applications. To this end, delivery systems capable of introducing the large prime editing and pegRNA components into primary cells will become instrumental.

## *In vivo* Delivery Options for Gene Editing Tools: From Classical AAV Vectors to Third-Generation AdV Vectors?

There are several methods to deliver a gene therapy to the retina. In general, these can be divided into viral and non-viral categories. Viral vectors consist of non-replicating viruses that carry the therapeutic genetic material that is introduced into target cells through transduction. Viral vectors are often used for CRISPR/Cas-mediated *in vivo* gene editing, however, delivering gRNAs and Cas nucleases directly as ribonucleoprotein complexes offers the distinct advantage of limiting the exposure of cells to long-lasting RGN activity (Kim et al., 2017; Lino et al., 2018; Yu and Wu, 2018). Non-viral vectors include lipid-based carriers or liposomes with chemical modifications (Chu-Tan et al., 2020), different nanoparticle formulations (Himawan et al., 2019) or salt solutions that enhance cell penetrance (iTOP) (D’Astolfo et al., 2015). For instance, cationic lipids were used to deliver Cas9 and gRNA as a ribonucleoprotein to the mouse retina following subretinal injection (Kim et al., 2017). In addition, synthetic nanoparticles with reduced cytotoxicity have been developed and tested for the *in vivo* delivery of RGN complexes to murine retinal pigment epithelium (Chen et al., 2019). Each method has its own set of advantages and limitations with regard to the treatment of retinal diseases. In this section, we will briefly discuss the use of AAV vectors, which are currently the gold standard vehicles for targeting the retina. Subsequently, we will focus on the potential use of HC-AdVs, also known as “gutless” or third-generation adenoviral vectors, to circumvent some of the limitations of the AAV-based platform.

AAV vectors are the most widely used tools for delivering genetic material to retinal cell types owing to their low immunogenicity and high transduction efficiencies (Trapani, 2019). They can be tested for gene replacement or gene editing therapies. Once inside the target cell nucleus, AAV vector genomes stay, for the most part, as extrachromosomal episomes that sustain long-term transgene expression in non-dividing cells. For recent reviews on the use of AAV vectors for gene therapy and on their optimization, including serotype selection and expression cassette design (e.g., promoter and transgene codon-optimization, see Wang et al., 2019; Li and Samulski, 2020). The tropism of the different AAV serotypes has been extensively characterized with regard to their capability to transduce different retinal cell types (e.g., photoreceptors, retinal pigment epithelium cells and Müller glial cells, amongst others). The different routes of AAV vector administration (e.g., intravitreal or subretinal) have equally been extensively characterized (e.g., Moore et al., 2018). Building upon these research efforts, AAV vectors are currently being tested in clinical trials for the treatment of several IRDs. These ongoing clinical developments have been recently summarized (i.e., Moore et al., 2018; Arbabi et al., 2019; Trapani, 2019; Vázquez-Domínguez et al., 2019).

As aforementioned, a major drawback of AAV vectors is their limited packaging capacity of ∼4.7 kb, that includes the two inverted terminal repeats. This limitation has been somewhat ameliorated with the development of so-called dual AAV vectors. The dual AAV vector strategy consists in splitting the sequence of a large transgene through two AAV vector genomes that, once inside the cell, can become concatemerized through recombination involving ITR sequences or shared homologous regions (McClements and Maclaren, 2017; Trapani, 2019). After this end-to-end recombination between the two AAV vector genomes, judiciously placed splice donor and splice acceptor motifs guarantee the assembly of full-length mRNA transcripts and ensuing expression of full-length gene products (McClements and Maclaren, 2017; Trapani, 2019). In the case of gene editing, different AAV vectors can be assembled for transferring expressing units encoding RGN components (i.e., Cas9 nucleases and gRNAs) or donor templates containing the exogenous DNA of interest (see, e.g., Chen and Gonçalves, 2016; Suzuki et al., 2016). However, the need for two or more AAV vectors increases the AAV product production burden, reduces the overall efficiency of the therapeutic approach and is expected to render regulatory approvals substantially more complex.

Still concerning the testing of AAV vectors for therapeutic gene editing, it is noteworthy mentioning recent data demonstrating prevalent integration of Cas9-encoding AAV DNA at RGN target sequences in animal models (Hanlon et al., 2019; Nelson et al., 2019). Thorough deep sequencing assays revealed that fragments of AAV ITRs, joined to full-length or truncated transgene sequences, were consistently found at RGN target sites in various mouse tissues, i.e., muscle, brain and cochlea (Hanlon et al., 2019). In addition to raising concerns linked to the chromosomal integration of RGN-coding sequences in transduced cells, these data extend previous and recent studies reporting that AAV vector DNA can become “captured” through homology-independent recombination processes at ZFN- and RGN-induced DSBs in the liver and eye, respectively, of mouse models (Anguela et al., 2013; Maeder et al., 2019). Collectively, these findings highlight the need to carefully monitor the accuracy of AAV-based gene editing procedures and stress the importance of expanding the range of gene editing vehicles, especially those that, similarly to AAV vectors, do not carry viral genes.

Albeit less often than AAV vectors, other viral vectors, such as lentiviral (LV) vectors and adenoviral (AdV) vectors, have also been tested for gene replacement in retinal degeneration. Gene therapy clinical trials using LV vectors have been initiated to deliver large transgenes encoding *ABCA4* and *MYO7A* (NCT01367444 and NCT01505062, respectively). However, the long-term outcome of these clinical trials is still pending. Prototypic AdV vectors based on the human adenovirus serotype 5 use the Coxsackie B and adenovirus receptor (CAR) to enter cells (Bergelson, 1997). This receptor is not detected in the outer segments of photoreceptors in mouse and human retina, while being detected in other retinal cells including retinal pigmented epithelium and Müller glial cells (Mallam et al., 2004). In this regard, ongoing research efforts are directed at modifying the tropism of AdV vectors by designing variants of the viral capsid or of the receptor-interacting fiber motifs (Gao et al., 2019; Tasca et al., 2020). Although still controversial, studies indicate that photoreceptor transduction in mouse retina can be achieved by deleting the RGD domain from the penton base capsomer of AdV serotype 5 (Mallam et al., 2004; Sweigard et al., 2010). In addition, AdV vector genomes are maintained as extrachromosomal elements in post-mitotic cells, offering the potential for stable and long-term transgene expression (Rauschhuber et al., 2012; Ricobaraza et al., 2020; Tasca et al., 2020). Moreover, when compared to free-ended viral and non-viral vector DNA, protein-capped AdV genomes have been shown to be refractory to chromosomal DNA integration in transduction experiments comprising the delivery of TALENs and RGNs (Holkers et al., 2014).

These findings on the amenability of AdV systems to tropism modifications and prevalent episomal nature, make investigating HC-AdV vector delivery of RGN machineries *in vitro* and *in vivo* appealing. In contrast to their first- and second-generation counterparts, these third-generation AdV vectors are devoid of all viral genes which expands their cargo capacity to 36 kb while reducing cytotoxicity and immune responses in transduced cells and animal models, respectively (Gonçalves and de Vries, 2006; Ricobaraza et al., 2020; Tasca et al., 2020). Their large cargo capacity has in fact permitted the delivery of all RGN components, including gRNA multiplexing formats, in single HC-AdV vector particles (Ehrke-Schulz et al., 2017; Brescia et al., 2020; Tasca et al., 2020). The capability of HC-AdV vectors to achieve long-term transgene expression *in vivo* has been demonstrated in the retina (Kreppel et al., 2002; Chen et al., 2008) and in other organs of small and large animal models (for a recent review, see Ricobaraza et al., 2020). Assessment of the potential of HC-AdV vectors for gene editing therapies is at early stages. For instance, HC-AdV-mediated delivery of high-specificity RGN pairs targeting a mutational hotspot in the dystrophin-encoding *DMD* gene, whose mutations underlie Duchenne muscular dystrophy, permitted restoring the *DMD* reading frame in muscle progenitor cells (Brescia et al., 2020). Upon triggering myogenic differentiation of the resulting *DMD*-edited cell populations, shorter yet partially functional, dystrophin molecules (i.e., Becker-like dystrophins) were readily detected (Brescia et al., 2020). In another study, Xia and colleagues investigated the use of HC-AdV vectors for the treatment of cystic fibrosis, a monogenic disease caused by mutations in the *CFTR* gene. They reported the seamless integration of the *CFTR*-encoding transgene at a predefined genomic locus in airway epithelial cells (Xia et al., 2018). For a recent review on the application of AdV systems for gene editing purposes; see Tasca et al., 2020.

## Combining Gene Editing With Cell-Based Approaches to Treat Retinal Degeneration

In this last section, we discuss cell-based approaches for the treatment of retinal degeneration. As neurodegenerative conditions, IRDs lead to a progressive loss of functional cell populations that cannot be replenished due to a lack of progenitors. Cell therapies for treating IRDs, are still highly experimental. These cell therapies are based on the transplantation into the retina of photoreceptor progenitors or retinal pigmented epithelium derived from either embryonic stem cells (ESCs) (Lamba et al., 2009; Shirai et al., 2016; Da Cruz et al., 2018) or induced pluripotent stem cells (iPSCs) (Reichman et al., 2017; Gagliardi et al., 2018). Cell therapies can be broadly divided in allogeneic and autologous. The former relies on the transplantation of cells from a healthy donor with HLA matching alleles; the latter involves the transplantation of patient-own cells previously genetically corrected *ex vivo*. Hence, transplantation of genetically corrected autologous cells integrates genetic and cell-based therapies. A recent study showed the feasibility in combining *ex vivo* correction of isolated photoreceptors with their subsequent transplantation into the retina in a murine model of retinitis pigmentosa (Barnea-Cramer et al., 2020). Recipient mice harbored a homozygous null mutation in the rhodopsin gene (*Rho*) that was corrected in isolated photoreceptor precursor cells through gene replacement using minicircle DNA delivery (Barnea-Cramer et al., 2020). Subsequently, these photoreceptor precursors were transplanted into the retina of mutant mice where they expressed maturation markers (e.g., *Pde6b* coding for phosphodiesterase 6 beta) at 3 months post-transplantation and, importantly, formed synaptic networks within the host retina (Barnea-Cramer et al., 2020). In fact, with the exception of electro retinal function, visual function and behavioral light responses were improved in recipient mutant mice, as measured by optomotor responses, behavioral light avoidance and pupil light reflex (Barnea-Cramer et al., 2020). In an *in vitro* study, the most prevalent mutations in the *USH2A* gene, responsible for Usher syndrome, characterized by retinitis pigmentosa and hearing loss, were corrected in patient-derived iPSCs by using enhanced specificity Cas9 from *S. pyogenes* (Sanjurjo-Soriano et al., 2020). Using whole-exome sequencing, no off-target events were detected after RGN-mediated *USH2A* correction. Moreover, the gene-edited iPSC lines retained their pluripotency as demonstrated by the expression of pluripotency markers SOX2, NANOG and OCT3/4, as well the ability to differentiate into the three germ layers, i.e., ectoderm, mesoderm and endoderm (Sanjurjo-Soriano et al., 2020).

Although far from its implementation in clinical trials, these reports highlight the possibility to integrate gene and cell therapy concepts and hence broaden the range of therapeutic options to halt retinal degeneration. There are nonetheless safety and efficacy concerns related to the source and differentiation capabilities of the transplanted cells. Most preeminent among these concerns is whether uncontrolled proliferation of iPSC-derived cells can occur and whether the graft will properly integrate in the host retina as to provide for functional rescue of the pathologic phenotype. Finally, in addition to the aforementioned assessments of the specificity and accuracy of gene editing procedures, much more research is warranted to (i) assure good manufacturing practices during the generation of retinal progenitors and (ii) implement exhaustive quality control protocols that minimize contaminants in cell products that may otherwise trigger adverse reactions.

## Conclusion

Many patients suffering from IRDs are still lacking therapeutic options that tackle the underlying cause of their blindness. There are many challenges on the path to the development of such therapies given the fact that, amongst the scientific and technological bottlenecks described herein, IRDs present considerable clinical and genotypic heterogeneity. In this review, we have presented some of the milestones and advances in gene editing strategies that are offering novel paths for treating IRDs. We have highlighted innovative gene editing concepts directed at achieving efficient and targeted chromosomal integration of therapeutic genes or precise *in situ* correction of genetic mutations in post-mitotic cells, such as those found in the retina. Therefore, it is plausible to consider that these novel gene editing strategies will start to be increasingly tested in *in vivo* models of retinal degeneration (e.g., mouse models) or in *in vitro* 3D human retinal organoids. Furthermore, although AAV vectors have demonstrated to be safe, well tolerated and efficient in delivering genetic material to the retina, they might not be the most suitable vehicle for transferring gene editing tools based on various CRISPR/Cas systems due to their limited packaging capacity and significant chromosomal integration potential. In conclusion, assisted by the implementation of delivery systems, future research avenues will undoubtedly focus on developing and optimizing gene editing components and procedures aiming at significantly improving the quality of life of patients afflicted by IRDs.

## Author Contributions

CG, MG, and JW performed the conceptualization and wrote, reviewed, and edited the manuscript. CG wrote the original draft. JW acquired the funding. All authors contributed to the article and approved the submitted version.

## Conflict of Interest

The authors declare that the research was conducted in the absence of any commercial or financial relationships that could be construed as a potential conflict of interest.

## Footnotes

1 https://sph.uth.edu/retnet/
